# An open-access WebApp for inverse Laplace transform analysis of time-domain nuclear magnetic resonance signals

**DOI:** 10.5194/mr-7-39-2026

**Published:** 2026-04-23

**Authors:** Tiago B. Moraes, Gustavo V. Von Atzingen, Larissa P. Mazzero, William S. Mendes, Marina B. Zacharias, Marcelo C. B. Cardinali

**Affiliations:** 1 Universidade de São Paulo/ESALQ, Depto. Engenharia de Biossistemas, Av. Páduas Dias, 11, 13418-900, Piracicaba, SP, Brazil; 2 Instituto Federal de Educação, Ciência e Tecnologia de São Paulo, Câmpus Piracicaba, Rua Diácono Jair de Oliveira, 1005, 13414-155, Piracicaba, SP, Brazil

## Abstract

Over recent years, compact and low-field time-domain nuclear magnetic resonance (TD-NMR) instruments have become increasingly available, expanding their use in the characterization of biomaterials across food, plant, and agro-industrial research. In this context, the inverse Laplace transform (ILT) has emerged as a powerful mathematical approach for extracting relaxation time distributions from TD-NMR signals. However, despite its widespread use, ILT analysis is often restricted to proprietary software or requires advanced expertise in numerical methods, limiting its accessibility to non-specialist users. In this work, we present an open-access WebApp for performing ILT analysis of TD-NMR signals in a transparent and user-friendly manner. The implemented algorithm is based on non-negative least squares combined with Tikhonov regularization and singular value decomposition, allowing robust inversion of ill-posed relaxation data. The platform supports the main TD-NMR experiments used in practice, including Carr–Purcell–Meiboom–Gill (CPMG), inversion recovery, and saturation recovery pulse sequences, and is compatible with data from instruments of any manufacturer. In addition to describing the mathematical formulation and implementation of the algorithm, a concise methodological discussion of ILT in the context of TD-NMR is provided. The performance of the WebApp is evaluated using both simulated datasets and representative experimental signals, demonstrating that the obtained relaxation time distributions are consistent with those produced by established ILT approaches. By lowering the barrier to advanced signal processing, the proposed WebApp represents a useful open scientific tool for research and teaching in magnetic resonance applications.

## 1 Introduction

The two best-known nuclear magnetic resonance (NMR) techniques in academia and industry are magnetic resonance imaging (MRI) and high-resolution NMR (HR-NMR). The former is widely applied in radiology for non-invasive medical diagnostics, whereas the latter is a well-established analytical technique used in chemical analysis laboratories for the precise characterization of molecular structures, playing a fundamental role in the pharmaceutical industry (Jacobsen, 2007; Blümich et al., 2014).

Time-domain NMR (TD-NMR), also referred to as low-field NMR (LF-NMR) or low-resolution NMR (LR-NMR) (Mitchell et al., 2014; Zalesskiy et al., 2014), is based on the same physical principles as MRI and HR-NMR but employs simpler instrumentation based on permanent magnets, without the need for large superconducting systems or cryogenic liquids. As a result, TD-NMR sensors and spectrometers have reduced size and cost, enabling the development of portable and benchtop instruments. These characteristics make TD-NMR a versatile technique for industrial and applied research, where it has increasingly been employed as a powerful analytical sensor (van Duynhoven et al., 2010; Song, 2013; Cheng et al., 2019; Caputo et al., 2019; Marassi et al., 2023; Azachi and Wiesman, 2026). Recent advances in electronics and permanent magnet technology have further contributed to the reduction of production and maintenance costs, fostering the emergence of several companies dedicated to compact TD-NMR instrumentation, such as Bruker, Magritek, Oxford, Nanalysis, SpinLock, FIT, and Niumag (Bruker, 2023; Magritek, 2023; Oxford, 2023; Nanalysis, 2023; SpinLock, 2023; FIT, 2023; Niumag, 2023).

For materials of biological origin, such as plants, foods, oils, seeds, and other agro-industrial products, magnetic resonance offers a non-destructive and nucleus-selective analytical approach, enabling the development of highly specific sensing methods. Among the available nuclei, ${}^{1}H$ detection is the most commonly employed due to its high sensitivity, although the use of other nuclei has steadily increased in low-field NMR applications as instrumentation has continued to improve (Blümich, 2016).

A wide variety of NMR methods can be implemented by applying different radio-frequency (rf) pulse sequences, resulting in distinct signal responses that encode diverse physicochemical information (Williams, 2005). The analysis of these signals is typically performed using mathematical approaches available in spectrometer software, including the Fourier transform (FT); exponential fitting; the inverse Laplace transform (ILT); and, more recently, machine learning techniques (Engelsen and van den Berg, 2017). Despite these developments, advanced signal-processing algorithms – particularly ILT – are often restricted to proprietary software or require specialized expertise, motivating many users to seek alternative implementations in environments such as C++, Python, MATLAB, or OriginLab (Moraes, 2021).

The objective of this paper is to present an open-access WebApp (https://nmr-ilt.esalq.usp.br/, last access: 15 April 2026) for the application of the inverse Laplace transform (ILT) to time-domain NMR (TD-NMR) signals, together with a concise methodological discussion of the underlying principles. The implemented algorithm is based on a non-negative least squares approach combined with Tikhonov regularization and singular value decomposition (SVD), enabling robust inversion of ill-posed relaxation data. In contrast to our previous ILT implementation reported in the literature (Moraes, 2021), which focused on local computational routines and specific applications, the present work provides a platform-independent, freely accessible web-based framework suitable for TD-NMR data from instruments of any manufacturer. The platform supports the main TD-NMR experiments used in practice, including Carr–Purcell–Meiboom–Gill (CPMG), inversion recovery, and saturation recovery pulse sequences. The main features of the proposed approach are validated using simulated datasets and representative experimental signals, demonstrating its consistency with established ILT methodologies and its potential as an open scientific tool for research and teaching in magnetic resonance applications.

## 2 Magnetic resonance background and motivation for ILT

Magnetic resonance techniques can be broadly distinguished by magnetic field strength and homogeneity, which directly determine signal characteristics and the appropriate data analysis strategy. High-field NMR systems employ highly homogeneous magnetic fields, enabling frequency-domain analysis with high spectral resolution through the Fourier transform (FT). In contrast, time-domain and low-field NMR (TD-NMR/LF-NMR) systems are typically based on permanent magnets, resulting in compact, cost-effective, and robust instrumentation but often with limited magnetic field homogeneity.

In many TD-NMR and benchtop systems, magnetic field inhomogeneities prevent reliable frequency-domain resolution, making relaxometry-based approaches more suitable. Pulse sequences such as Carr–Purcell–Meiboom–Gill (CPMG), inversion recovery (IR), and related methods are therefore widely used to probe longitudinal ( $T_{1}$ ) and transverse ( $T_{2}$ ) relaxation processes. The signals acquired using these sequences consist of superpositions of exponentially decaying components, whose amplitudes and characteristic times reflect molecular dynamics and local interactions within the sample.

From a signal-processing perspective, relaxometry data represent an inverse problem in which the measured signal is modeled as a weighted sum of exponential decays. Unlike the Fourier transform, which provides a direct and well-posed transformation, the determination of relaxation time distributions from TD-NMR signals constitutes an ill-posed inverse problem. In such cases, small perturbations in the input data – such as noise or experimental uncertainty – can lead to large variations in the recovered solution, necessitating the use of constrained optimization and regularization strategies.

Within this context, the inverse Laplace transform (ILT) has emerged as a powerful framework for analyzing TD-NMR relaxometry data. Practical implementations of the ILT rely on numerical algorithms that balance stability, resolution, and robustness through appropriate regularization and non-negativity constraints. These methodological aspects are central to the present work and motivate the development of a transparent, accessible, and reproducible platform for ILT-based analysis of TD-NMR signals, as described in the following section.

## 3 Inverse Laplace transform

In time-domain and low-field NMR experiments, signal analysis is predominantly performed in the time domain, particularly through relaxometry-based methods. In heterogeneous systems, the measured signal generally reflects a superposition of contributions arising from distinct spin populations characterized by different relaxation times, leading to multi-exponential signal behavior.

The present work does not aim to review relaxometry techniques in detail, which are extensively discussed elsewhere (Mitchell et al., 2014; Zalesskiy et al., 2014; Maus et al., 2006), but instead focuses on the mathematical processing of multi-exponential signals acquired using pulse sequences such as Carr–Purcell–Meiboom–Gill (CPMG), inversion recovery (IR), saturation recovery (SR), and related methods.

In simple systems, relaxation data can often be adequately described by mono-exponential models. However, in more complex samples, the measured signal is better represented as a superposition of multiple exponential components, each associated with a distinct relaxation time. In such cases, conventional multi-exponential fitting becomes unstable and strongly dependent on the assumed number of components.

In these situations, the analysis of relaxation data is commonly formulated as an inverse problem and addressed using inverse Laplace transform (ILT) methodologies, which aim to recover continuous distributions of relaxation times from experimental signals (Mitchell et al., 2014; Jacobsen, 2007; Moraes, 2021).

**Figure 1:**
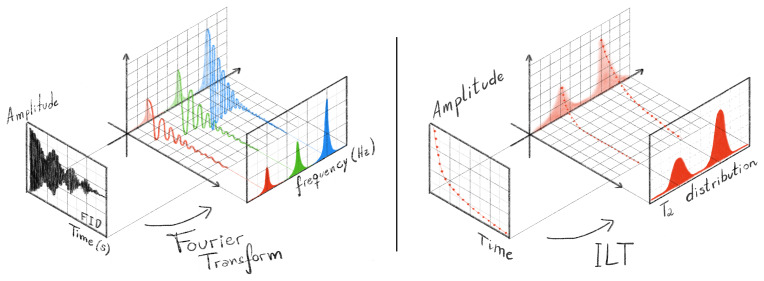
Comparative schematic illustration of time-domain NMR signal processing using the Fourier transform (FT) and the inverse Laplace transform (ILT). In the FT approach (left panel), which is the most widely known and routinely used method in NMR spectroscopy, the free induction decay (FID) consists of a mixture of oscillatory, exponentially damped sinusoids in the time domain. After Fourier transformation, these components are separated in the frequency domain, producing a spectrum whose peak positions, line widths, and amplitudes provide chemical and structural information about the sample. In contrast, the ILT approach (right panel) is applied to non-oscillatory signals. On the left side of this panel, a Carr–Purcell–Meiboom–Gill (CPMG)-type signal is represented by the echo amplitudes as a function of time, showing purely monotonic exponential decays rather than oscillations. By processing this signal with the ILT, the result is not a frequency spectrum but a distribution of relaxation times ( $T_{2}$ ), shown on the right, also called a relaxogram. The positions and areas of the $T_{2}$ peaks reflect physical and compositional properties of the analyzed material, such as pore size distribution, water and oil content, and meat-to-fat ratio. Together, the figure highlights the conceptual analogy between both transforms – each mapping time-domain signals into characteristic distributions – while emphasizing the fundamental difference between oscillatory (FT) and non-oscillatory (ILT) data.

Figure 1 schematically illustrates the objective of ILT processing, in which a time-domain relaxometry signal is transformed into a relaxation time distribution. The positions and areas of the resulting peaks reflect the relative contributions of different spin populations present in the sample.

In this way, the ILT process reveals the contribution of different spin groups, where the two wide peaks illustrated in the $T_{2}$ distribution can be a better representation of a real sample once real samples are not straightly homogeneous with singular values of amplitude and relaxation times (narrow peaks), but they are actually inhomogeneous samples, with a dispersion of relaxation times.

The distribution obtained in the processing with the ILT can arise from a growing or decaying exponential damped curve, as with CPMG ( $T_{2}$ ) or inversion recovery ( $T_{1}$ ), saturation recovery ( $T_{1}$ ), diffusion ( D ), among other experiments, where what changes in the processing is the kernel equation used in the transformation, as will be discussed in the next section.

There are several software platforms to perform this processing, some with free access and others only being supplied with licenses by the spectrometer manufacturing companies, each with different processing characteristics. The most famous ILT algorithms are CONTIN (CONTINuous distribution) (Provencher, 1982), UpenWin (Uniform PENalty) (Borgia et al., 1998), BRD (Butler–Reeds–Dawson) (Butler et al., 1981), TSVD (truncated singular value decomposition) (Fordham et al., 2017), NNLS (non-negative least squares) (Lawson and Hanson, 1976), PDCO (primal–dual interior method) (Berman et al., 2013), among bi-dimensional ILT (Venkataramanan et al., 2002; Bortolotti et al., 2019) and other methods.

In the next section, the basic concepts of the ILT and a simple-to-use WebApp developed for performing the transformation of TD-NMR signals are presented, freely available for research and teaching. A mathematical description of the implemented processing is then provided, followed by results with simulated and experimental data and, finally, the procedures for using the program. A video tutorial demonstrating the code and its usage is available on the WebApp page or can be requested from the authors by email.

### Mathematical description of the ILT

There are several mathematical approaches to perform the inversion of these TD-NMR signals, and these techniques are generally referred to as inverse Laplace transform by the NMR scientific community. It is worth noting that the method discussed here is different from the procedure of the same name found in mathematical physics books (Arfken and Weber, 1995). Despite the confusion with the name, the origin of the uses of this term by the NMR community is related to an analogy with the procedure of the inverse Fourier transform. Further details of this history can be found in the work of Fordham and colleagues (Fordham et al., 2017).

In the context of TD-NMR, for most of the pulse sequences used, the acquired signals show exponential and/or Gaussian growth or decay. The inversion problem to be solved here is to determine all of the amplitudes and relaxation times present in the data. Therefore, what we call ILT is a particular case of a more general problem of inverting Fredholm integral equations, which can be written in the following form:

1 $$f(x)=\int_{a}^{b}\phi (t)\;K(x,t)\;dt,$$

where K(x,t) is the kernel function of the system, and the function ϕ(t) represents the function to be determined from the experimental data curve f(x) .

In the case of an experimental signal from a CPMG (Carr–Purcell–Meiboom–Gill) pulse sequence, the model function F(t) is given by $F(t)=c(0)\;\mathrm{exp}(-t/T_{2})$ , where c(0) is the initial amplitude of the experimental signal, t is the time variable of the acquired signal, and $T_{2}$ is the relaxation time. For a sample composed of multiple relaxation times, we have the following:

2 $$F(t)=\sum_{i=1}^{k}c_{i}(0)\;\mathrm{exp}(-t/T_{2i}),$$

where k denotes the number of components present in the sample.

In real complex samples, it is physically expected not to have only a singular value of relaxation time but rather to have a continuous distribution of relaxation times. Thus, we can express the NMR signal as given by the following integral:

3 $$F(t)=\int g(T_{2})\;\mathrm{exp}(-t/T_{2})\;dT_{2},$$

where $\mathrm{exp}(-t/T_{2})$ is the kernel function, and $g(T_{2})$ represents the distribution of relaxation times $T_{2}$ , providing the amplitudes of each infinitesimal component $dT_{2}$ .

The ILT algorithm will determine the distribution of amplitudes $g(T_{2})$ from the experimental data $c(t_{n})$ ; however, experimental NMR signals $c(t_{n})$ are a set of n data points acquired over the discrete time points $t_{n}$ , with the presence of experimental noise $\epsilon (t_{n})$ ; thereby

4 $$c(t_{n})=\sum_{i=1}^{N}\;g(T_{2i})\;K(t_{n},T_{2i})+\epsilon (t_{n}),$$

where K is the kernel equation of this summation. Note that N denotes the total points of the $T_{2}$ dimension, which need to be defined in a window of expected relaxation times, for example, from 0.001 to 10 s using N=100 or 200 points. For a CPMG experiment, the kernel should be defined as a composition of decreasing exponential, as shown in Eq. (2). The inversion of this problem can be obtained through the minimization of the mean square errors, given by

5 $$X^{2}=\|c(t_{n})-F(t_{n}){\|}^{2},$$

where $F(t_{n})$ is the model function that best describes the experimental data $c(t_{n})$ . This minimization problem is not trivial as it involves a mathematically ill-posed problem (Istratov and Vyvenko, 1999; Venkataramanan et al., 2002; Tikhonov, 1963).

Several regularization methods can be used to obtain solutions for this Fredholm integral problem, each presenting different characteristics of stability and reliability. Physically, in our TD-NMR experiments, it is reasonable to expect that our solution $g(T_{2})$ is a continuous distribution of relaxation times without the existence of delta peaks, discontinuous points, or negative values of amplitude in the distribution (Fig. 1). These constraints can be introduced into the program's algorithm through a term that smooths the distribution and enforces non-negativity, as proposed by the NNLS (non-negative least squares) method. This regularization of the solution enforces more stable and continuous distributions, and the regularization parameter ( α ) is responsible for the smoothing of this solution.

Different values of the regularization parameter α can yield optimal curve fits, resulting in distributions with certain variations. Determining which distribution best represents the physical system is not trivial and, therefore, involves some subjectivity as the fit strongly depends on the signal-to-noise ratio of the data. Various methods have been used and proposed to optimize the choice of the regularization parameter α , such as the L-curve method (Hansen, 1992; Day, 2011). In the present implementation, no automatic optimization strategy for the regularization parameter α is employed. Instead, α is selected by the user based on prior knowledge of the system and the signal-to-noise characteristics of the data, an approach commonly adopted in practical ILT analyses. Future versions of the WebApp will incorporate automated strategies for α selection.

Therefore, the algorithm of the WebApp uses the Tikhonov regularization method with singular value decomposition while assuming the non-negativity of the response, also known as NNLS (non-negative least squares), widely used by the NMR community.

## 4 ILT-NMR WebApp

An open-access WebApp was developed to enable inverse Laplace transform (ILT) analysis of TD-NMR data without the need for local software installation. The application is deployed using Docker and hosted on secure servers of the University of São Paulo (USP) within the InterNuvem infrastructure, ensuring stable and reliable access for the scientific community. The computational core of the WebApp was implemented in Python (Van Rossum and Drake Jr., 1995), making use of established numerical and visualization libraries, including NumPy (Harris et al., 2020) and Plotly. The implemented algorithm represents a platform-independent adaptation of our previously reported ILT routine developed for OriginLab software (Moraes, 2021).

The WebApp supports ILT processing of relaxation data acquired using Carr–Purcell–Meiboom–Gill (CPMG), inversion recovery (IR), and saturation recovery (SR) pulse sequences. For each experiment type, the corresponding kernel function K(t,T) is defined as follows:

6 $$\begin{array}{rl} & \text{CPMG: }K(t,T)=[\mathrm{exp}(-t/T)],\\ & \text{IR: }K(t,T)=[1-2\;\mathrm{exp}(-t/T)],\\ & \text{SR: }K(t,T)=[1-\mathrm{exp}(-t/T)], \end{array}$$

where t denotes the experimental time axis, and T represents the relaxation time domain of the resulting ILT distribution.

**Figure 2:**
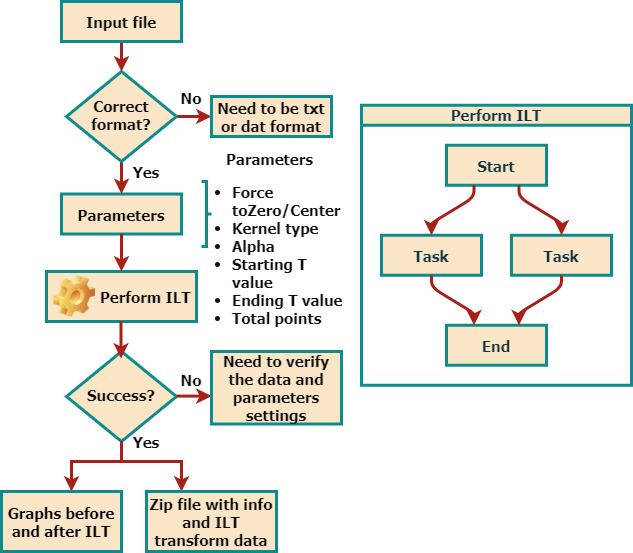
WebApp pipeline.

Figure 2 summarizes the data-processing workflow implemented in the WebApp. Input signals are provided as text-based files containing the experimental time axis and one or more corresponding signal amplitudes. After specifying the experiment type and processing parameters, the ILT inversion is performed, and the resulting relaxation time distributions are returned in graphical and numerical formats, allowing direct inspection and further analysis. The uploaded data are temporarily processed on the server and are automatically discarded after the analysis, without being stored or logged.

The WebApp interface was designed to provide flexible control over key processing parameters, including the definition of the relaxation time window, discretization of the T axis, baseline correction, signal normalization, and the choice of the regularization parameter α . While the present implementation requires manual selection of α , this approach allows the user to incorporate prior knowledge of the sample and signal-to-noise characteristics into the analysis. Automated strategies for α optimization will be incorporated into future versions of the platform. To facilitate reproducibility and broader use, the WebApp accepts multiple signals sharing a common time axis, enabling batch processing and comparative analysis of TD-NMR experiments.

**Figure 3:**
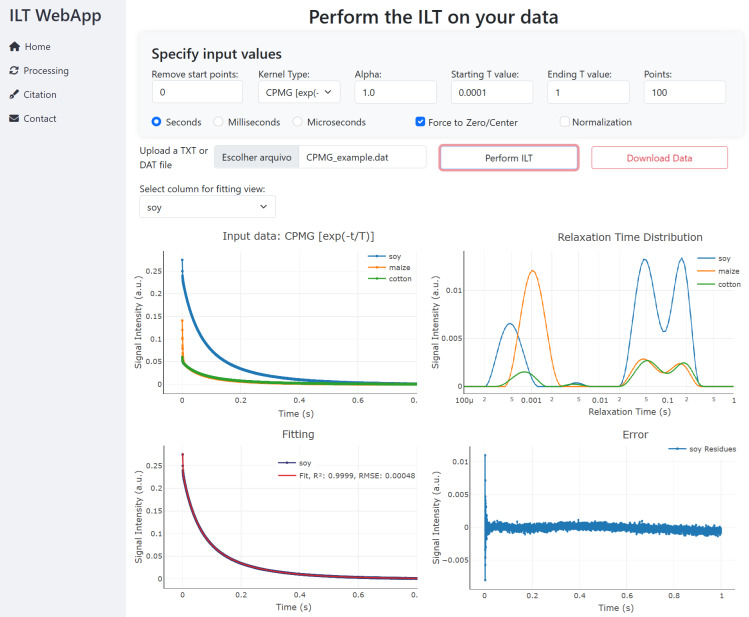
WebApp simplified processing interface.

Figure 3 shows the Processing page of the user interface, available at: https://nmr-ilt.esalq.usp.br/ (last access: 15 April 2026). The full navigation bar on the left includes Home (main page with general information about the ILT process), Processing (the core section for data processing), Citation, and Contact (to send a message to the authors).

In the Processing bar, it is required that information be provided regarding the input parameters and information:

- *Kernel type.* Information regarding what type of data the user is processing must be provided. There are three options, namely CPMG, IR, and SR kernels, as shown in Eq. (6).
- *Remove start points.* If desired, some initial points of the data can be removed. It is useful when delaying with spectrometer *dead-time* effects or spurious data points.
- *Alpha.* The regularization parameter α is always necessary to be defined, and it is related to the *resolution* of the spectrum. Usually, the larger the α value, the lower spectral resolution; however, the forcing resolution can provide unreliable spectra in data with a low signal-to-noise ratio. In this WebApp, its value is defined by the user. Typical values used are 0.01, 0.1, 1, and 10.
- *Starting T value.* Set the minimum value of the relaxation times expected, in the timescale defined.
- *Ending T value.* Set the maximum value of the relaxation times expected, in the timescale defined.
- *Number of points.* Define the total number of points in the resulting distribution spectrum. Typical values are 100 or 200.
- *Timescale.* It must be defined whether the time axis is in seconds, milliseconds, or microseconds. After processing, the relaxation time T axis in the ILT spectra will be of the same order of magnitude.
- *Force to zero/center.* Spurious peaks can arise from a small displacement of the baseline of the input signals (offset), and this function can be used to perform this pre-processing step.
- *Normalization.* This function will divide the data in each column by the first value of the signal, thus normalizing the signals.
- *Perform ILT.* Once the processing is initiated, in a few seconds (or minutes if many columns are provided), the results are presented in the figures. An option to download all of the images and data points is also available.

**Figure 4:**
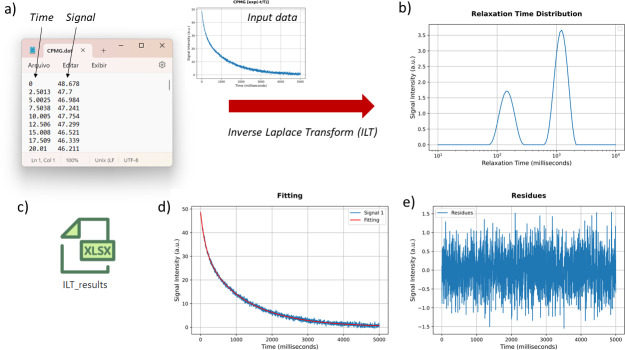
The signal input needed to have a minimum of two columns: first, the time axis and, second, the amplitude signal c(t) . The results of ILT processing are presented in panels **(b)** to **(e)**, with **(b)** the relaxation time distribution, **(d)** the comparison of the fitting and input signal, and **(e)** its residues. All data results can be downloaded in an xlsx file.

Figure 4 presents an example of the input data format and the corresponding ILT processing results, including the relaxation time distribution, fitting, residuals, and exported data. The input data must be provided in a .txt or .dat file, where the first column represents the time axis, and the second contains the signal intensity. Multiple signals can also be processed simultaneously by placing them in the subsequent columns, provided they share the same time axis (first column). In this case, all signals will be processed with the same input parameters, which is convenient for comparison and analysis of dynamic process experiments. The first row may optionally be used to define the axis labels.

After the ILT processing, resulting figures are generated in the WebPage. Figure 4b shows the relaxation time distribution obtained, with the x axis in logarithmic scale, (d) shows the comparison between the *fitting* and the input signal, and (e) its respective residues. All data and figures can be downloaded in a zip file.

Three example files with experimental data are available on the Home page. The ILT WebApp can be accessed at https://nmr-ilt.esalq.usp.br (last access: 15 April 2026), and a video tutorial demonstrating its use is available at https://www.youtube.com/watch?v=_n_sN_G3Cnk (last access: 15 April 2026).

## 5 Results and discussions

To evaluate the performance and robustness of the implemented ILT algorithm, a series of tests were conducted using simulated and experimental TD-NMR signals acquired under different conditions for CPMG, inversion recovery (IR), and saturation recovery (SR) experiments. The results presented below focus on the influence of regularization parameters, the fidelity of the recovered relaxation time distributions, and the consistency of the results with known ground-truth distributions and literature data.

### 5.1 Influence of the regularization parameter α

**Figure 5:**
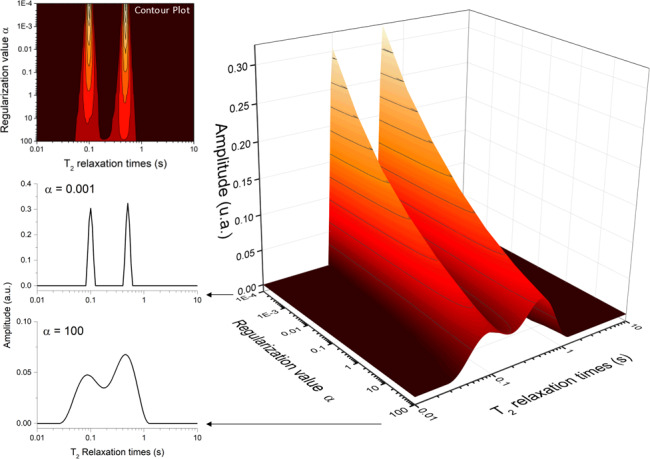
Effect of the regularization parameter ( α ) on the resulting relaxation distribution spectrum. The larger the value of α , the broader the peaks become.

Figure 5 illustrates the effect of the regularization parameter α on the relaxation time distribution obtained from the ILT of a bi-exponential CPMG signal with relaxation times of 100 and 500 ms. As expected, increasing α leads to broader distributions with reduced peak amplitudes while preserving the total spectral area. This behavior reflects the trade-off between resolution and stability that is characteristic of regularized inverse problems.

The choice of α is particularly critical for signals with a limited signal-to-noise ratio, where excessive regularization may oversmooth physically relevant features, whereas insufficient regularization can amplify noise-induced artifacts. In practice, the selection of α is guided by balancing these two effects, aiming to minimize spurious oscillations while preserving meaningful spectral features.

A common strategy is to select the smallest α value that yields a stable and physically interpretable distribution, without the appearance of noise-driven peaks. In this context, visual inspection of the resulting distribution, combined with knowledge of the expected number of components and their approximate relaxation times, can provide a practical and effective criterion.

Although automated optimization strategies such as the L-curve method have been proposed in the literature (Hansen, 1992; Day, 2011), the present implementation allows manual selection of α , enabling the incorporation of prior knowledge about the sample and experimental conditions. This flexibility is particularly useful in heterogeneous systems, where the definition of an optimal α may depend on the specific analysis objective, such as resolution of closely spaced components or suppression of noise artifacts.

### 5.2 Validation using simulated and experimental signals

**Figure 6:**
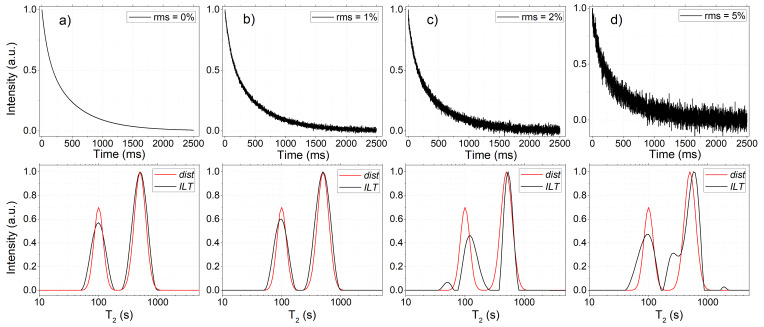
Simulated CPMG signals were generated to evaluate the performance of the ILT WebApp. Panels **(a)**–**(d)** show signals with increasing rms noise levels, obtained by numerical integration of a predefined relaxation time distribution (shown below as dist, red line). The original distribution consists of two peaks centered at relaxation times of 100 and 500 ms. After processing the time-domain data using the ILT algorithm, the recovered distributions (ILT, black line) are presented below each signal. Several similar simulated datasets were analyzed, and the results were found to be consistent with those obtained using established ILT software.

The algorithm was first validated using simulated TD-NMR signals generated from predefined relaxation time distributions. Broad log-Gaussian distributions were used to emulate realistic heterogeneous systems, and synthetic CPMG and IR signals were obtained by numerical integration of these distributions, followed by the addition of Gaussian white noise at different rms levels. Figure 6a–d present representative simulated signals with increasing noise levels, corresponding to rms values of 0 %, 1 %, 2 %, and 5 %, respectively. For each case, the corresponding ILT distribution obtained from the simulated signal is shown below, alongside the original distribution used to generate the signal, allowing a direct comparison between the recovered and true distributions.

From 0 % to 2 % rms, the recovered distributions show good agreement with the ground-truth distributions used in the simulations, accurately reproducing peak positions, widths, and relative amplitudes. The relative deviation of peak centers remained within a few percent for all tested cases, consistently with previously reported results for established ILT implementations. As the noise level increases from 2 % to 5 % rms, the recovered ILT distributions begin to exhibit spurious peaks, reflecting the inherent ill-posed nature of the ILT problem in the presence of noise, as widely documented.

**Figure 7:**
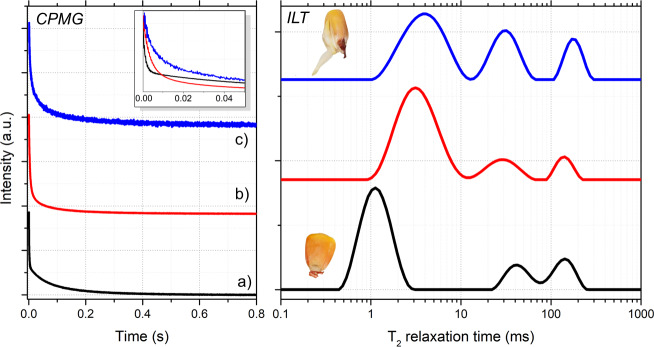
Comparison of CPMG signals (left) and the corresponding relaxation time distributions (right) of maize seeds under different conditions: (a) dry seeds with moisture content below 10 %; (b) seeds after 24 h of water absorption; and (c) seeds after several days, when germination had begun, as illustrated in the inset above. These results are consistent with Song et al. (2022)'s analysis of maize seeds (Song et al., 2022).

Beyond simulations, the algorithm was applied to experimental TD-NMR data acquired from maize seed samples at different hydration and germination stages (Fig. 7). The ${}^{1}H$ TD-NMR measurements were performed on an 11.3 MHz spectrometer (0.27 T for ${}^{1}H$ resonance frequency), SLK-200 (SpinLock, Argentina), using a 30 mm probe at 30 °C . The Carr–Purcell–Meiboom–Gill (CPMG) pulse sequence was used, with 90 and 180° pulses of 9.0 and 18.5 µs , respectively, and an echo time of 200 µs , with a total of 4000 echoes, a recycle delay of 3 s, and 32 scans. For ILT processing, α=1 and 100 points were used.

The three ILT spectra of Fig. 7 are from a set of nine maize seeds measured simultaneously: first, in (a), measured when they are dry with less than 10 % moisture; in (b), measured after the set of maize seeds absorbed water by 24 h; and, in (c), after some days, when they started germination, as showed in the insert illustration of a maize seed. It can be noted in (a) that the main peak, at first at $T_{2}=1$ ms, shifted to 3 ms in (b) and also got wider. Also, the two smaller peaks in (a), around 40 and 150 ms, shift and widen in (b). These changes reflect the process of water absorption and the increase in heterogeneity inside the maize seeds. These processes can be monitored in more detail and studied to interpret what each peak represents inside the maize seed. These peaks of $T_{2}$ relaxation times in seeds typically represent signals from different portions of water and/or oil mobility due the porous structures.

In Fig. 7c, the ILT spectrum of germinated maize seeds is presented, as shown in the inset illustration of a maize seed. These resulting relaxation time distributions reveal systematic shifts and broadening of the $T_{2}$ peaks as water uptake and metabolic activity increase, reflecting changes in molecular mobility and structural heterogeneity within the seeds. These trends are consistent with previously reported TD-NMR studies of maize seeds and validate the applicability of the proposed approach to real agro-biological systems (Song et al., 2022).

Taken together, these results demonstrate that the implemented ILT algorithm reliably recovers relaxation time distributions from both simulated and experimental TD-NMR data, producing results that are consistent with established methods. Moreover, the same core algorithm has been successfully applied by our research group in a wide range of previous studies, including food analysis (Moraes and Colnago, 2022), meat aging and color evaluation (Cônsolo et al., 2021; Moreira et al., 2016), physiological disorders in poultry and fruits (Cônsolo et al., 2020; Bizzani et al., 2020), seed and plant science (Monaretto et al., 2021), enzymatic activity in cassava roots (da Silva Ferreira et al., 2018), and the detection of counterfeit spirits (de Lima et al., 2023), further supporting its robustness and practical applicability.

### 5.3 Tips and tricks

**Figure 8:**
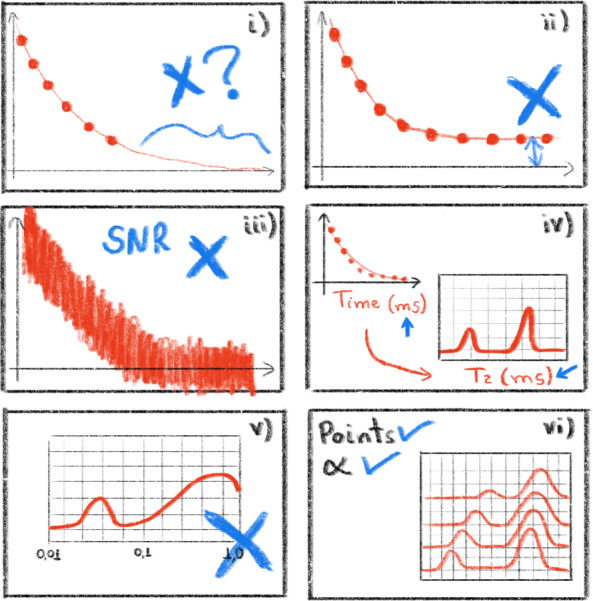
Illustration with tips and tricks that must be observed when processing signals with the ILT WebApp. Panel **(i)** shows the problem of discontinuity experimental data signal, panel **(ii)** shows offset amplitude, panel **(iii)** shows the signal-to-noise ratio, panel **(iv)** shows the time axis, panel **(v)** shows the selection of an expected window of relaxation times, and panel **(vi)** shows the uses of the same parameters values to compare several signals. Figure adapted from reference (Moraes, 2021).

To ensure accurate analysis of the ILT processing, certain practical aspects need to be emphasized, as illustrated in Fig. 8:

i. Signals with exponential decay and/or growth should be fully acquired during the experimental stage, with a sufficiently long baseline. Discontinuities in the initial points or a step in the final baseline, as in illustration (i), can lead to artificial (spurious) peaks in the resulting ILT spectrum.
ii. Artificial peaks may arise in the ILT spectrum from slight displacements of the baseline (amplitude offset). To address this, the WebApp provides the option of a pre-processing function, namely “Force to zero/center”, for this correction.
iii. The ILT spectrum is strongly influenced by the signal-to-noise ratio of the acquired data. Noisy experimental signals can result in unreliable distributions.
iv. Note that the ILT spectrum will have the same time unit as the input signal. We recommend using times in seconds or milliseconds for the input signal as relaxation times of biologic samples typically fall within the range of 0.001 and 10 s.
v. Note that the ILT spectrum window (starting T value and ending T value) and the number of points (100) must be selected carefully. If the window is too short or too long, it can generate distributions with artificial and distorted peaks.
vi. For studies comparing several signals, it is advisable to consistently use the same parameter values in both the experimental acquisition stage and the ILT processing. For the ILT processing, these parameters include the number of points, the α parameter, and the values of the starting T value and ending T value.

By paying attention to these practical considerations, more accurate and meaningful ILT analyses can be achieved.

## 6 Conclusion

This work presented the fundamental principles of the inverse Laplace transform (ILT) applied to time-domain NMR (TD-NMR) data and introduced an open-access WebApp designed to perform this analysis in a transparent and platform-independent manner. The implemented approach is based on non-negative least squares combined with Tikhonov regularization, providing a robust framework for the inversion of ill-posed relaxometry problems commonly encountered in TD-NMR experiments.

Validation using both simulated datasets with known ground-truth distributions and representative experimental signals demonstrated that the proposed implementation reliably recovers relaxation time distributions, yielding results consistent with those obtained using established ILT software. These findings confirm the methodological soundness of the algorithm and support its use as a complementary tool for TD-NMR data analysis in research and educational contexts.

The WebApp is freely accessible online at https://nmr-ilt.esalq.usp.br/ (last access: 15 April 2026), offering an accessible environment for ILT analysis without the need for proprietary software. Future developments will focus on extending the available functionalities and incorporating automated strategies for regularization parameter selection.

## Data Availability

Code is not publicly available because it is implemented within a web-based platform that is freely accessible to users. The source code is not distributed in order to ensure appropriate use within the intended platform and to avoid unauthorized commercial reuse. Data are available from the authors upon request.

## Sample availability

Samples are available from the authors upon request.

## Video supplement

A video tutorial demonstrating the use of the WebApp is available at https://youtu.be/_n_sN_G3Cnk (Moraes, 2026).
